# The Association Between Bronchoscopy and the Prognoses of Patients With Ventilator-Associated Pneumonia in Intensive Care Units: A Retrospective Study Based on the MIMIC-IV Database

**DOI:** 10.3389/fphar.2022.868920

**Published:** 2022-06-08

**Authors:** Luming Zhang, Shaojin Li, Shiqi Yuan, Xuehao Lu, Jieyao Li, Yu Liu, Tao Huang, Jun Lyu, Haiyan Yin

**Affiliations:** ^1^ Department of Intensive Care Unit, The First Affiliated Hospital of Jinan University, Guangzhou, China; ^2^ Department of Clinical Research, The First Affiliated Hospital of Jinan University, Guangzhou, China; ^3^ Department of Orthopaedics, The First Affiliated Hospital of Jinan University, Guangzhou, China; ^4^ Department of Neurology, The First Affiliated Hospital of Jinan University, Guangzhou, China; ^5^ Guangdong Provincial Key Laboratory of Traditional Chinese Medicine Informatization, Guangzhou, China

**Keywords:** ICU, ventilator-associated pneumonia, bronchoscopy, mortality, causal mediation analysis

## Abstract

**Background:** In intensive care units (ICUs), the morbidity and mortality of ventilator-associated pneumonia (VAP) are relatively high, and this condition also increases medical expenses for mechanically ventilated patients, which will seriously affect the prognoses of critically ill patients. The purpose of this study was to determine the impact of bronchoscopy on the prognosis of patients with VAP undergoing invasive mechanical ventilation (IMV).

**Methods:** This was a retrospective study based on patients with VAP from the Medical Information Mart for Intensive Care IV database. The outcomes were ICU and in-hospital mortality. Patients were divided based on whether or not they had undergone bronchoscopy during IMV. Kaplan-Meier (KM) survival curves and Cox proportional-hazards regression models were used to analyze the association between groups and outcomes. Propensity score matching (PSM) and propensity score based inverse probability of treatment weighting (IPTW) were used to further verify the stability of the results. The effect of bronchoscopy on prognosis was further analyzed by causal mediation analysis (CMA).

**Results:** This study enrolled 1,560 patients with VAP: 1,355 in the no-bronchoscopy group and 205 in the bronchoscopy group. The KM survival curve indicated a significant difference in survival probability between the two groups. The survival probabilities in both the ICU and hospital were significantly higher in the bronchoscopy group than in the no bronchoscopy group. After adjusting all covariates as confounding factors in the Cox model, the HRs (95% CI) for ICU and in-hospital mortality in the bronchoscopy group were 0.33 (0.20–0.55) and 0.40 (0.26–0.60), respectively, indicating that the risks of ICU and in-hospital mortality were 0.67 and 0.60 lower than in the no-bronchoscopy group. The same trend was obtained after using PSM and IPTW. CMA showed that delta-red blood cell distribution width (RDW) mediated 8 and 7% of the beneficial effects of bronchoscopy in ICU mortality and in-hospital mortality.

**Conclusion:** Bronchoscopy during IMV was associated with reducing the risk of ICU and in-hospital mortality in patients with VAP in ICUs, and this beneficial effect was partially mediated by changes in RDW levels.

## Introduction

Ventilator-associated pneumonia (VAP), defined as infection of the lung parenchyma in patients after at least 48 h of exposure to invasive mechanical ventilation (IMV), (Papazian et al., 2020), is one of the most common infectious diseases in intensive care units (ICUs) (Hunter, 2012), affecting up to 40% of patients on mechanical ventilators (Spalding et al., 2017). Various previous studies found that VAP was associated with longer IMV durations and ICU stays, and also increased antimicrobial use (Hayashi et al., 2013). In some developed countries, VAP was also found to increase the average hospitalization cost of patients by approximately US$ 40,000 (Zimlichman et al., 2013). The mortality rate of patients with VAP may exceed 50% (Ruiz et al., 2000); the results of 58 randomized studies on VAP indicated that the estimated attributable mortality rate was 9% (range 3–17%) (Melsen et al., 2011). VAP has high morbidity and mortality, and also increases the medical expenses of mechanically ventilated patients, which will seriously affect the prognoses of critically ill patients.

Bronchoscopy has been widely used for clinical diagnoses and treatment of respiratory diseases. Airway examination and transbronchial biopsy have greatly improved the diagnosis rates of pulmonary inflammatory (Baselski and Wunderink, 1994; Su et al., 2020) and substantial lung (Navani et al., 2011) diseases, and can also help improve the treatment effect (Pedro et al., 2019). However, currently there is no unified conclusion on whether bronchoscopy can improve the prognoses of patients with VAP. This study used the large public Medical Information Mart for Intensive Care (MIMIC)-IV database as a basis to determine the impact of bronchoscopy on the prognoses of patients with VAP in the ICU.

## Methods

### Data Source and Population

The MIMIC-IV is a large, free, and open database; the latest version is MIMIC-IV (version 1.0) (Johnson et al., 2021), which contains comprehensive information on approximately 250,000 patients hospitalized from 2008 to 2019, and provides strong data support for clinical studies (Yang et al., 2020; Wu et al., 2021). The database was approved by the Massachusetts Institute of Technology (Cambridge, Mass.) and the Beth Israel Deaconess Medical Center (Boston, Mass.), and consent was obtained for collection of the original data (Zhang et al., 2021). The database also anonymizes patient information, and so informed consent did not need to be obtained. The researchers needed to complete corresponding courses and obtain certificates to access and extract data from this database.

ICU patients diagnosed with VAP in the MIMIC-IV database were included. For patients admitted to the ICU more than once, only information on the first admission was obtained. Patients without a detailed record of ventilator use or who stayed in the ICU for less than 24 h were excluded. Patients were divided based on whether or not they had undergone bronchoscopy during IMV.

### Data Extraction

Data with less than 20% missing values in the database were extracted using Structured Query Language. The included demographic information were age, sex, body mass index (BMI), and race. Other information included the first care unit, disease severity [ Acute Physiology Score III (APSIII)], interventional therapy [vasopressor use, continuous renal replacement therapy (CRRT) use, and duration of IMV], major comorbidities [sepsis, myocardial infarction (MI), congestive heart failure (CHF), hypertension, cerebrovascular disease (CD), chronic pulmonary disease (CPD), liver disease (LD), renal disease (RD), diabetes, and malignancy]; microbiology; results of the first laboratory tests after ICU admission [white blood cells (WBC), neutrophils, lymphocytes, basophils, eosinophils, monocytes, red blood cells (RBC), hematocrit, hemoglobin, mean corpuscular volume (MCV), mean corpuscular hemoglobin (MCH), mean corpuscular hemoglobin concentration (MCHC), red blood cell distribution width (RDW), platelets, anion gap (AG), bicarbonate, calcium total, calcium free, magnesium phosphate, chloride, sodium, potassium, base excess (BE), calculated total CO_2_, pH, PaCO_2_, PaO_2_, lactate, creatinine, blood urea nitrogen (BUN), glucose, international normalized ratio (INR), prothrombin time (PT), partial thromboplastin time (PTT), aspartate aminotransferase (AST), alanine aminotransferase (ALT), alkaline phosphatase (AP), lactate dehydrogenase (LDH), total bilirubin, and albumin], and vital signs within 24 h of ICU admission [mean heart rate (mHR), mean value of mean arterial pressure (mMAP), mean respiratory rate (mRR), mean temperature (mT), mean SpO2(mSpO2), urine output].

The outcomes of this study were ICU and in-hospital mortality.

### Statistical Analyses

In this study, Pearson’s correlation coefficient was adopted to determine the correlation between characteristic variables and to remove strongly correlated variables (Zhang et al., 2022). The following variables had coefficients higher than 0.6, so were removed since they were considered strongly correlated: RBC, hematocrit, MCV, chloride, BE, calculated total CO_2_, pH, BUN, PT, AST, and LD (Supplementary Figure S1). For other variables with less than 20% of missing values, the “mice” package of R software was used to perform multiple imputation.

The data for continuous variables were represented as mean and standard-deviation or median and interquartile (IQR) values, while those for categorical data were represented as frequencies. The Mann-Whitney U test was used for continuous variables, and the χ^2^ test or Fisher’s exact test was used for categorical variables.

The Kaplan-Meier (KM) method draws a cumulative incidence curve, indicating the occurrence of ICU and in-hospital deaths in different groups of patients, and the differences in risk between the groups were compared using log-rank tests. Furthermore, after adjusting different covariates, two Cox proportional-hazards models were constructed to analyze the influence of the relationship between bronchoscopy and outcomes. There were no adjustments for covariates in model I. In model II, all variables, including age, sex, race, BMI, first care unit, APSIII, vasopressors, CRRT, duration of IMV, sepsis, MI, CHF, hypertension, CD, CPD, LD, RD, diabetes, malignancy, WBC, neutrophils, lymphocytes, basophils, eosinophils, monocytes, hemoglobin, MCH, MCHC, RDW, platelet, AG, bicarbonate, calcium total, calcium free, magnesium phosphate, sodium, potassium, PaCO2, PaO2, lactate, creatinine, glucose, INR, PTT, ALT, AP, bilirubin total, albumin, mHR, mMAP, mRR, mT, mSpO2, urine output were adjusted for confounding factors. In the multivariate COX regression, we also evaluated the multicollinearity between the variables using variance inflation factors (VIF). Supplementary Table S1 revealed that the VIF of each variable was less than 4, indicating that there was no multicollinearity between them.

To ensure that the results were stable and reliable, we further adjusted for covariates using propensity score matching (PSM) and propensity score-based inverse probability of treatment (IPTW) after analyzing the original population. Multivariate logistic regression model was used to estimate patient propensity scores by using one-to-one nearest neighbor matching with a caliper width of 0.05. The IPTW model was created using estimated propensity scores as weights. Differences in baseline levels between the two groups were evaluated using *p* values. Then, COX regression was performed on the matched population and the weighted population, respectively.

Causal mediation analysis (CMA) can distinguish the total effect of treatment into direct effects and indirect effects, If the independent variable X has a certain influence on the dependent variable Y through a certain variable M, then M is called the mediating variable of X and Y (Imai et al., 2010). In the present study, we hypothesized that changes in a particular indicator were mediating variables, that is, we assumed that bronchoscopy might lead to changes in that indicator and that such changes were associated with the prognosis of VAP patients. The average causal mediating effect (ACME), average direct effect (ADE) and total effect obtained through CMA can help us verify the above conjecture. Finally, WBC, platelets and RDW, three indicators that were further investigated for the presence of mediating effects because of their low absence rate after the cessation of IMV. Their change level was expressed as the difference between the corresponding first examination results and recorded as delta-WBC, delta-RDW, delta-platelet.

We also analyzed the impact of bronchoscopy on the prognoses of patients from different subgroups. The subgroups included age (dichotomized at 65 years), sex, type of first care unit, APSIII (dichotomized at the median of 69), and all comorbidities. The interactions between subgroups were further analyzed.

A two-tailed probability value of *p* < 0.05 was considered statistically significant. All statistical analyses in this study were performed using R software (version 4.1.0).

## Results

### Baseline Characteristics

**FIGURE 1:**
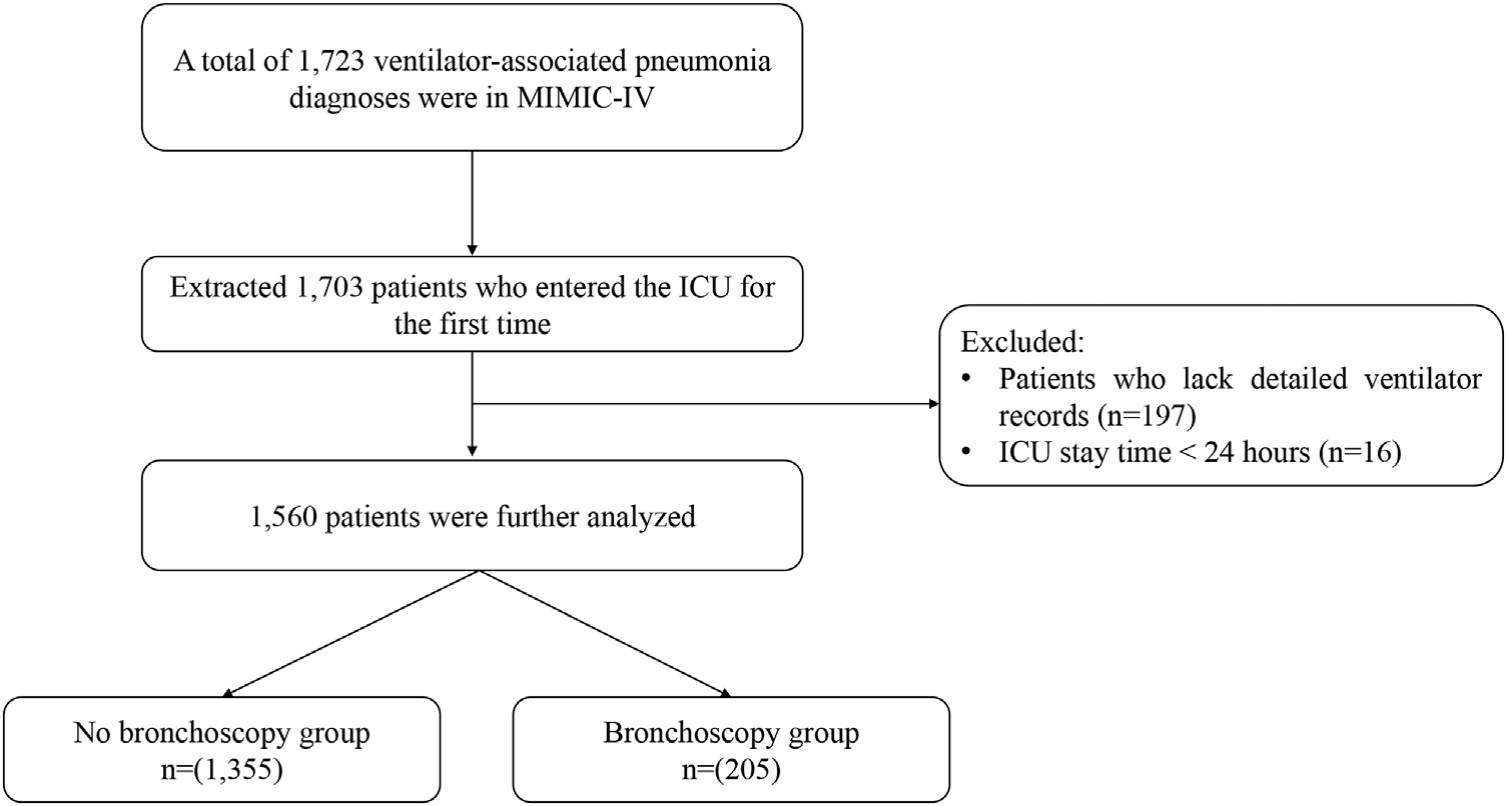
Inclusion and exclusion flowchart of the study.

This study enrolled 1,560 patients with VAP: 1,355 in the no-bronchoscopy group and 205 in the bronchoscopy group (Figure 1). The median (IQR) ages of the patients in these two groups were 64.00 years (53.00–75.00 years) and 62.00 years (50.00–74.00 years), respectively; there were more male patients in the two groups (62.7 and 65.9%, respectively); the median (IQR) APSIII were 69.00 (51.00–90.00) and 70.00 (56.00–91.00), respectively; the median (IQR) durations of IMV were 136.00 h (60.50–253.50 h) and 145.00 h (59.00–286.00 h), respectively; and sepsis was the most common complication, present in 96.5 and 97.6% of patients in the two groups, respectively. The remaining baseline characteristics of the patients are listed in detail in Table 1.

**TABLE 1:** Baseline characteristics of original population.

	Original Population			PSM Population		
	No Bronchoscopy Group	Bronchoscopy Group	*p*-Value	No Bronchoscopy Group	Bronchoscopy Group	*p*-Value
N	1,355	205		195	195	
Age (year)	64.00 (53.00, 75.00)	62.00 (50.00, 74.00)	0.302	66.00 (53.50, 76.00)	63.00 (50.00, 75.50)	0.546
Gender (%)			0.432			0.915
Male	850 (62.7)	135 (65.9)		129 (66.2)	127 (65.1)	
Female	505 (37.3)	70 (34.1)		66 (33.8)	68 (34.9)	
Ethnicity (%)			0.177			0.139
White	772 (57.0)	130 (63.4)		105 (53.8)	123 (63.1)	
Black	154 (11.4)	17 (8.3)		17 (8.7)	17 (8.7)	
Others	429 (31.7)	58 (28.3)		73 (37.4)	55 (28.2)	
BMI	28.25 (24.25, 33.69)	28.44 (25.60, 34.66)	0.184	29.01 (24.39, 35.15)	28.40 (25.55, 34.61)	0.844
APSIII	69.00 (51.00, 90.00)	70.00 (56.00, 91.00)	0.286	71.00 (57.00, 94.50)	71.00 (56.00, 91.00)	0.395
First care unit (%)			0.001			0.426
MICU/SICU	1,007 (74.3)	174 (84.9)		157 (80.5)	164 (84.1)	
Others	348 (25.7)	31 (15.1)		38 (19.5)	31 (15.9)	
Duration of IMV (hour)	136.00 (60.50, 253.50)	145.00 (59.00, 286.00)	0.332	127.00 (64.50, 243.50)	145.00 (56.50, 287.00)	0.306
Vasopressors (%)	518 (38.2)	63 (30.7)	0.046	61 (31.3)	60 (30.8)	1.000
CRRT (%)	43 (3.2)	5 (2.4)	0.726	5 (2.6)	5 (2.6)	1.000
Comorbidities						
Sepsis (%)	1,308 (96.5)	200 (97.6)	0.578	192 (98.5)	190 (97.4)	0.721
Myocardial infarct (%)	257 (19.0)	24 (11.7)	0.015	34 (17.4)	24 (12.3)	0.200
Congestive heart failure (%)	453 (33.4)	59 (28.8)	0.214	63 (32.3)	55 (28.2)	0.440
Hypertension (%)	625 (46.1)	101 (49.3)	0.444	93 (47.7)	97 (49.7)	0.761
Cerebrovascular disease (%)	346 (25.5)	47 (22.9)	0.474	54 (27.7)	46 (23.6)	0.417
Chronic pulmonary disease (%)	388 (28.6)	70 (34.1)	0.125	62 (31.8)	66 (33.8)	0.746
Liver disease (%)	255 (18.8)	33 (16.1)	0.401	41 (21.0)	33 (16.9)	0.366
Renal disease (%)	308 (22.7)	37 (18.0)	0.157	38 (19.5)	36 (18.5)	0.897
Diabetes (%)	430 (31.7)	48 (23.4)	0.02	52 (26.7)	45 (23.1)	0.482
Malignancy (%)	127 (9.4)	23 (11.2)	0.478	21 (10.8)	22 (11.3)	1.000
Vital signs						
Mean heart rate (min-1)	86.54 (74.81, 99.31)	87.09 (75.58, 98.89)	0.701	84.37 (73.52, 97.26)	87.37 (75.65, 99.66)	0.202
Mean Mbp (mmHg)	77.64 (70.94, 85.10)	76.16 (71.04, 84.00)	0.545	75.88 (69.81, 82.98)	76.16 (70.98, 83.57)	0.309
Mean respiratory rate (min-1)	19.71 (17.39, 22.67)	19.73 (17.34, 22.76)	0.646	20.12 (17.44, 23.03)	19.73 (17.40, 22.83)	0.450
Mean temperature (°C)	37.07 (36.69, 37.45)	36.95 (36.60, 37.37)	0.107	37.07 (36.67, 37.42)	37.00 (36.60, 37.37)	0.392
Mean SpO2 (%)	97.93 (96.19, 99.11)	97.63 (95.96, 98.81)	0.069	97.86 (96.15, 98.97)	97.64 (96.04, 98.78)	0.438
Urineoutput (ml)	1,490.00 (859.00, 2315.00)	1,620.00 (915.00, 2285.00)	0.811	1,425.00 (819.50, 2132.50)	1,620.00 (902.50, 2292.50)	0.317
Microbiology (%)			0.120			0.058
Unclear	413 (30.5)	53 (25.9)		55 (28.2)	46 (23.6)	
Gram positive	251 (18.5)	39 (19.0)		23 (11.8)	37 (19.0)	
Gram negative	531 (39.2)	96 (46.8)		88 (45.1)	95 (48.7)	
Others	160 (11.8)	17 (8.3)		29 (14.9)	17 (8.7)	
Laboratory tests						
WBC (k/uL)	11.80 (8.60, 16.40)	12.20 (9.40, 16.10)	0.464	11.80 (8.40, 16.90)	12.30 (9.50, 16.30)	0.498
Neutrophils (%)	81.00 (74.00, 86.50)	84.00 (76.70, 88.50)	<0.001	83.00 (76.05, 88.10)	84.00 (76.15, 88.25)	0.625
Lymphocytes (%)	9.20 (5.70, 14.70)	8.60 (5.40, 13.00)	0.171	9.80 (5.60, 15.00)	8.70 (5.40, 13.10)	0.310
Basophils (%)	0.20 (0.00, 0.40)	0.20 (0.00, 0.30)	0.557	0.20 (0.00, 0.40)	0.20 (0.00, 0.30)	0.356
Eosinophils (%)	0.60 (0.00, 1.80)	0.60 (0.10, 1.40)	0.891	0.40 (0.00, 1.50)	0.60 (0.10, 1.40)	0.171
Monocytes (%)	5.50 (3.60, 8.00)	4.60 (3.00, 6.00)	<0.001	4.60 (3.00, 6.00)	4.60 (3.00, 6.00)	0.936
Hemoglobin (g/dl)	10.60 (8.80, 12.50)	10.50 (9.40, 12.30)	0.282	11.00 (9.30, 12.65)	10.50 (9.40, 12.30)	0.543
MCH (pg)	30.20 (28.60, 31.65)	30.50 (29.10, 31.70)	0.107	30.70 (29.40, 31.80)	30.40 (29.05, 31.70)	0.322
MCHC (%)	32.70 (31.50, 33.80)	33.20 (31.80, 34.40)	<0.001	33.30 (32.10, 34.20)	33.20 (31.85, 34.40)	0.786
RDW (%)	14.60 (13.55, 16.30)	14.60 (13.60, 16.10)	0.737	14.60 (13.50, 15.90)	14.50 (13.60, 16.00)	0.849
Platelet (k/uL)	189.00 (133.50, 251.00)	204.00 (138.00, 277.00)	0.096	186.00 (146.50, 250.00)	204.00 (136.50, 275.50)	0.391
Anion Gap (mEq/L)	15.00 (12.00, 18.00)	14.00 (12.00, 17.00)	0.114	15.00 (12.00, 17.00)	14.00 (12.00, 17.00)	0.432
Bicarbonate (mEq/L)	22.00 (19.00, 25.00)	24.00 (21.00, 27.00)	<0.001	24.00 (21.00, 27.50)	24.00 (21.00, 27.00)	1.000
Total calcium (mEq/L)	8.20 (7.70, 8.80)	8.30 (7.80, 8.90)	0.062	8.40 (7.80, 8.90)	8.30 (7.80, 8.90)	0.791
Free calcium (mEq/L)	1.11 (1.04, 1.16)	1.11 (1.05, 1.17)	0.099	1.11 (1.05, 1.16)	1.11 (1.05, 1.17)	0.546
Magnesium (mEq/L)	1.90 (1.70, 2.20)	2.00 (1.80, 2.20)	0.105	1.90 (1.80, 2.20)	2.00 (1.80, 2.20)	0.694
Phosphate (mEq/L)	3.60 (2.90, 4.50)	3.70 (3.10, 4.60)	0.121	3.50 (2.90, 4.70)	3.60 (3.05, 4.55)	0.390
Sodium (mEq/L)	139.00 (136.00, 143.00)	140.00 (136.00, 142.00)	0.763	139.00 (137.00, 143.00)	140.00 (136.00, 142.00)	0.903
Potassium (mEq/L)	4.10 (3.70, 4.60)	4.10 (3.70, 4.50)	0.712	4.10 (3.70, 4.60)	4.10 (3.70, 4.50)	0.876
PaCO2 (mmHg)	41.00 (35.00, 50.00)	43.00 (37.00, 55.00)	0.004	43.00 (36.50, 51.50)	43.00 (37.00, 54.50)	0.981
PaO2 (mmHg)	106.00 (65.00, 192.00)	122.00 (84.00, 209.00)	0.006	127.00 (81.00, 218.00)	122.00 (84.00, 210.00)	0.956
Lactate (g/dl)	1.70 (1.10, 2.70)	1.50 (1.00, 2.70)	0.061	1.60 (1.15, 2.60)	1.50 (1.00, 2.70)	0.266
Creatinine (g/dl)	1.00 (0.80, 1.70)	1.00 (0.70, 1.60)	0.161	1.00 (0.80, 1.65)	1.00 (0.70, 1.60)	0.174
Glucose (mg/dl)	139.00 (111.00, 181.00)	131.00 (109.00, 168.00)	0.058	136.00 (111.00, 167.50)	133.00 (110.00, 168.00)	0.600
INR	1.30 (1.10, 1.50)	1.20 (1.10, 1.40)	0.217	1.30 (1.10, 1.50)	1.20 (1.10, 1.40)	0.208
PTT (s)	30.60 (26.90, 38.00)	30.80 (27.30, 37.70)	0.688	31.60 (27.20, 39.60)	30.80 (27.35, 37.60)	0.404
ALT (iu/L)	30.00 (17.00, 61.00)	34.00 (18.00, 67.00)	0.16	28.00 (17.00, 63.50)	34.00 (18.00, 69.50)	0.325
AP (iu/L)	78.00 (57.00, 114.00)	81.00 (54.00, 114.00)	0.911	76.00 (57.00, 108.50)	81.00 (54.50, 115.00)	0.692
Total bilirubin (mg/dl)	0.60 (0.40, 1.10)	0.60 (0.40, 1.10)	0.821	0.60 (0.40, 1.00)	0.60 (0.40, 1.10)	0.964
Albumin (g/dl)	2.90 (2.50, 3.30)	2.90 (2.60, 3.50)	0.309	3.00 (2.60, 3.40)	2.90 (2.60, 3.50)	0.334
Delta-WBC	-1.00 (-4.55, 2.60)	-1.20 (-5.20, 2.40)	0.488	-1.20 (-4.62, 1.83)	-1.15 (-5.40, 2.63)	0.934
Delta-RDW	0.50 (-0.10, 1.70)	0.30 (-0.30, 1.30)	0.046	0.60 (0.00, 1.50)	0.35 (-0.30, 1.40)	0.353
Delta-platelet	58.50 (-9.00, 176.25)	106.00 (-0.50, 208.50)	0.048	64.00 (-1.00, 177.00)	104.00 (-6.50, 208.75)	0.192

### Survival Analysis and Cox Proportional-Hazards Regression Model

**FIGURE 2:**
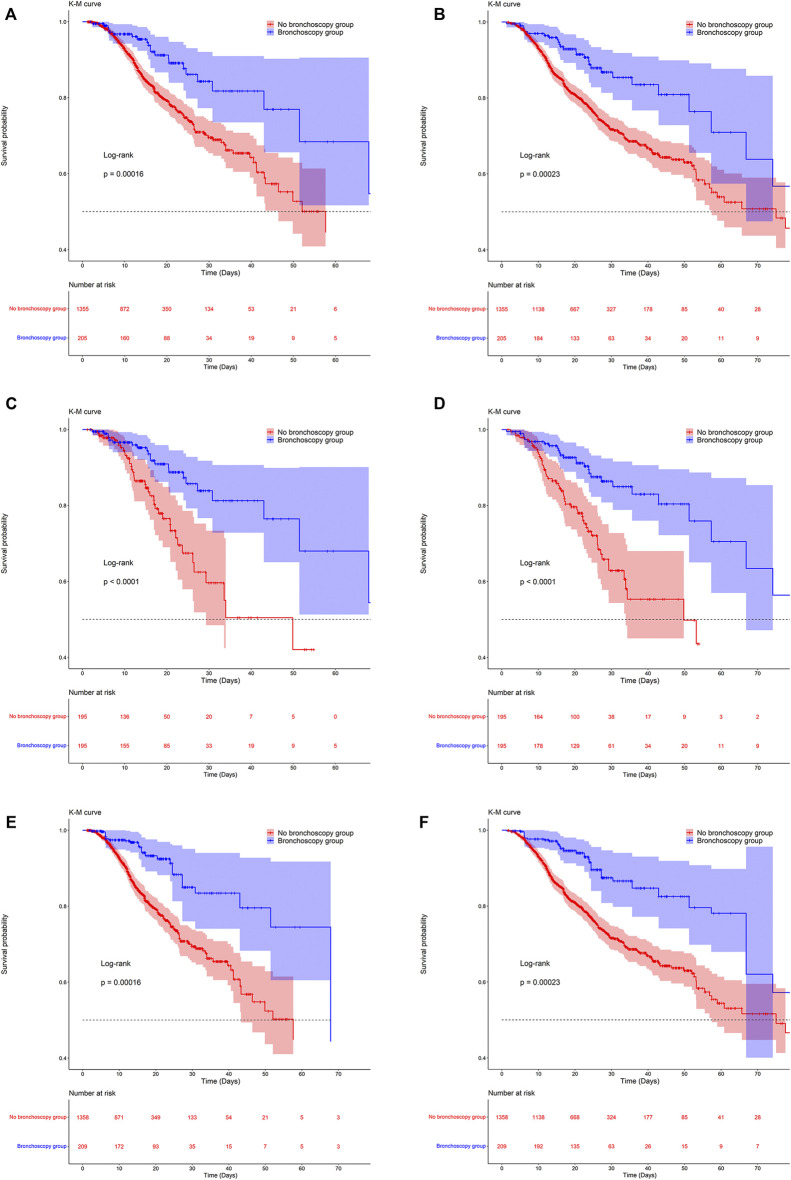
Kaplan-Meier survival curves between groups. **(A,C,E)** are the ICU mortality risk for the original population, the PSM population and the IPTW population; **(B,D,F)** are the in-hospital mortality risk for the original population, the PSM population and the IPTW population.

The KM survival curve in Figure 2 indicated a significant difference in survival probability between the two groups. The survival probability in the ICU and in hospital was significantly higher in the bronchoscopy group than in the no-bronchoscopy group (Figure 2). The results of the log-rank test indicated that the mortality risks in the ICU and in hospital differed between the two groups.

Table 2 lists the results for the Cox proportional-hazards model. In two models with no adjustment for confounders, or adjustment for all confounders, the hazard ratio (HR) was significantly lower than one for the bronchoscopy group compared with the no-bronchoscopy group. In other words, compared with patients in the no-bronchoscopy group, those in the bronchoscopy group had a lower risk of ICU and in-hospital mortality. In model II, after adjusting for all covariates as confounding factors, the HR (95% CI) values for ICU and in-hospital mortality in the bronchoscopy group were 0.33 (0.20–0.55) and 0.40 (0.26–0.60), respectively, indicating that the risks of ICU and in-hospital mortality were 0.67 and 0.60 lower than in the no-bronchoscopy group. (Table 2).

**TABLE 2:** Results of Cox proportional hazard models.

	Model I		Model II	
Outcomes	HR (95%CI)	*p*-Value	HR (95%CI)	*p*-Value
Original population				
ICU Mortality				
Bonchoscopy				
no	Reference		Reference	
yes	0.44 (0.29,0.68)	<0.001	0.33 (0.20,0.55)	<0.001
In-hospital Mortality				
Bonchoscopy				
no	Reference		Reference	
yes	0.49 (0.33,0.72)	<0.001	0.40 (0.26,0.60)	<0.001
After PSM				
ICU Mortality				
Bonchoscopy				
no	Reference		Reference	
yes	0.36 (0.21,0.61)	<0.001	0.33 (0.17,0.64)	0.001
In-hospital Mortality				
Bonchoscopy				
no	Reference		Reference	
yes	0.38 (0.24,0.59)	<0.001	0.33 (0.20,0.58)	<0.001
After IPTW				
ICU Mortality				
Bonchoscopy				
no	Reference		Reference	
yes	0.37 (0.23,0.60)	<0.001	0.26 (0.14,0.50)	<0.001
In-hospital Mortality				
Bonchoscopy				
no	Reference		Reference	
yes	0.42 (0.27,0.65)	<0.001	0.35 (0.21,0.57)	<0.001

### Propensity Score Matching and Inverse Probability of Treatment Weighing

After PSM and IPTW, there was no significant difference in baseline levels between the two groups (Supplementary Table S2). Kaplan-Meier survival curves of matched and weighted populations are consistent with the original population (Figure 2). Univariate and multivariate COX regressions were then performed on the matched and weighted populations, respectively, yielding results consistent with the original population (Table 2). After multivariate COX regression, HR (95% CI) values for ICU mortality in the bronchoscopy group were 0.33 (0.17,0.64) and 0.26 (0.14,0.50), and in-hospital mortality the HR (95% CI) values of 0.33 (0.20,0.58) and 0.35 (0.21,0.57), respectively.

### Causal Mediation Analysis

**FIGURE 3:**
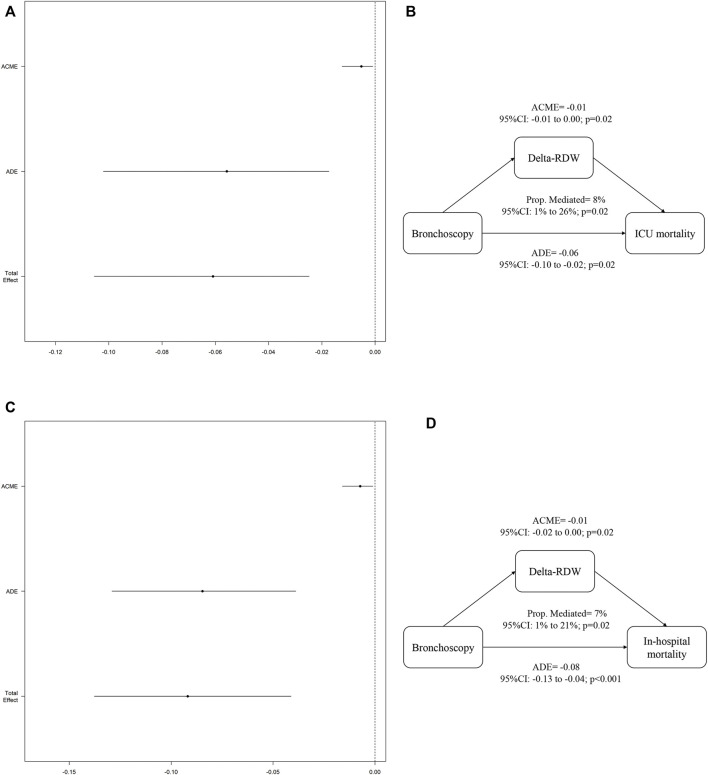
Causal mediation analysis for RDW reduction.

In the CMA analysis, after analyzing the three indicators, only the changes of RDW were significant. Figures 3A, B showed that in terms of in-hospital mortality of VAP patients, delta-RDW mediated 8% (95% CI:1–26%; *p* = 0.02) of the beneficial effect of bronchoscopy (ACME:*p* = 0.02). At the same time, Figures 3C, D implied that in terms of ICU mortality, delta-RDW mediated 7% (95% CI: 1–21%; *p* = 0.02) of the beneficial effects of bronchoscopy (ACME:*p* = 0.02).

### Subgroup Analysis

There was no significant difference between the subgroups of in-hospital mortality, meaning that no interaction was present. However, bronchoscopy was associated with a reduced risk of ICU mortality in MI and CPD populations, with HRs (95% CI) of 0.21 (0.05–0.86) and 0.13 (0.05–0.34), respectively. Meanwhile, patients without LD were more likely to benefit from bronchoscopy than those with LD (HR = 0.29, 95% CI = 0.16–0.54) (Table 3).

**TABLE 3:** Subgroup analysis of relationship between groups and mortality.

	ICU Mortality			Hospital Mortality		
	HR (95%CI)	*p*-value	p-interaction	HR (95%CI)	*p*-value	p-interaction
Age			0.773			0.442
<65 (*n* = 791)	0.61 (0.26,1.44)	0.261		0.59 (0.28,1.25)	0.167	
≥65 (*n* = 769)	0.28 (0.15,0.53)	<0.001		0.34 (0.20,0.57)	<0.001	
Gender			0.055			0.150
Male (*n* = 985)	0.23 (0.11,0.46)	<0.001		0.32 (0.18,0.55)	<0.001	
Female (*n* = 575)	0.36 (0.17,0.82)	0.014		0.47 (0.23,0.92)	0.029	
First care unit			0.941			0.576
MICU/SICU (*n* = 1,181)	0.32 (0.18,0.56)	<0.001		0.35 (0.22,0.57)	<0.001	
Others (*n* = 379)	0.30 (0.09,1.03)	0.056		0.44 (0.16,1.16)	0.097	
APSIII			0.381			0.390
<69 (*n* = 763)	0.49 (0.18,1.36)	0.170		0.40 (0.17,0.93)	0.032	
≥69 (*n* = 797)	0.30 (0.17,0.54)	<0.001		0.36 (0.22,0.59)	<0.001	
Microbiology			0.162			0.597
Gram positive (*n* = 290)	0.46 (0.08,2.49)	0.369		0.36 (0.11,1.20)	0.095	
Gram negative (n = 627)	0.27 (0.11,0.64)	0.003		0.35 (0.18,0.68)	0.002	
Sepsis						
No (*n* = 52)	NA			NA		
Yes (*n* = 1,508)	0.33 (0.20,0.55)	<0.001		0.39 (0.25,0.59)	<0.001	
Myocardial infarct			0.007			0.118
No (*n* = 1,279)	0.44 (0.26,0.75)	0.002		0.44 (0.28,0.70)	<0.001	
Yes (*n* = 281)	0.21 (0.05,0.86)	0.031		0.24 (0.07,0.82)	0.022	
Congestive heart failure			0.207			0.647
No (*n* = 1,048)	0.45 (0.24,0.84)	0.012		0.39 (0.23,0.67)	<0.001	
Yes (*n* = 512)	0.17 (0.07,0.42)	<0.001		0.31 (0.15,0.61)	<0.001	
Hypertension			0.317			0.725
No (*n* = 834)	0.22 (0.10,0.48)	<0.001		0.35 (0.20,0.63)	<0.001	
Yes (*n* = 726)	0.33 (0.16,0.68)	0.003		0.28 (0.14,0.56)	<0.001	
Cerebrovascular disease			0.240			0.223
No (*n* = 1,167)	0.28 (0.15,0.51)	<0.001		0.40 (0.25,0.64)	<0.001	
Yes (*n* = 393)	0.31 (0.09,1.02)	0.054		0.34 (0.13,0.89)	0.028	
Chronic pulmonary disease			0.004			0.151
No (*n* = 1,102)	0.55 (0.30,1.01)	0.053		0.48 (0.28,0.82)	<0.001	
Yes (n = 458)	0.13 (0.05,0.34)	<0.001		0.16 (0.07,0.36)	<0.001	
Renal disease			0.563			0.556
No (*n* = 1,215)	0.29 (0.16,0.54)	<0.001		0.36 (0.21,0.59)	<0.001	
Yes (*n* = 345)	0.36 (0.12,1.08)	0.069		0.50 (0.22,1.13)	0.096	
Liver disease			0.015			0.379
No (*n* = 1,272)	0.24 (0.13,0.45)	<0.001		0.36 (0.22,0.57)	<0.001	
Yes (*n* = 288)	0.64 (0.18,2.27)	0.491		0.56 (0.21,1.50)	0.246	
Diabetes			0.563			0.549
No (*n* = 1,082)	0.38 (0.21,0.68)	0.001		0.43 (0.27,0.69)	<0.001	
Yes (*n* = 478)	0.36 (0.11,0.94)	0.039		0.16 (0.07,0.36)	<0.001	
Malignant cancer						
No (n = 1,410)	0.25 (0.14,0.44)	<0.001		0.33 (0.21,0.53)	<0.001	
Yes (n = 150)	NA			NA		

## Discussion

Few previous studies have investigated the prognostic effects of bronchoscopy among patients with VAP, with many studying the value of bronchoscopy for diagnosing or preventing VAP (Timsit et al., 2001), or limited to specific populations such as trauma or pediatric patients (Bush, 2003; Nannapaneni et al., 2021). The present study included patients with VAP from the large public MIMIC-IV database as the study population. In this present study, we took the VAP patients in the large public database MIMIC-IV as the study population, whether it was univariate analysis, or adjusted for a number of confounding factors such as demography, intervention, disease severity score, complications, vital signs, laboratory examination indicators, etc., and reached a consistent conclusion, that is, in patients with IMV in ICU, the risk of ICU mortality and in-hospital mortality in VPA patients who underwent bronchoscopy were significantly lower than those who did not receive bronchoscopy. In addition, after PSM and IPTW, the trend was consistent with that of the original population, which proved that our results were robust and reliable.

We believe that most VAP patients in the ICU are critically ill patients with severe diseases such as sepsis (Markwart et al., 2020), with unclear self-consciousness (Hübscher and Isenmann, 2016) and poor sputum expectoration ability (Tang et al., 2011), causing large amounts of sputum or respiratory secretions cannot be discharged and block the trachea, aggravating the condition. At the same time, there are a large number of inflammatory factor secretions in the patient’s airway, forming a cascade amplification effect, which can spread to cause systemic inflammation (Zaragoza et al., 2020), which seriously affects the patient’s prognosis. While bronchoscopy can go deep into the lower respiratory tract and directly reach the lungs with the most severe disease (Estella, 2012). When patients are undergoing IMV, bronchoscopy can not only help suck out the obstructions such as sputum and foreign bodies in the trachea, remove secretions, but also repeatedly suction and wash the lungs, which is of great significance for reducing inflammation and improving lung ventilation.

RDW is a parameter that reflects the heterogeneity of red blood cells in the blood, and is also a new marker of inflammation (Hou et al., 2018). At present, there are a lot of published evidences indicating that there is an inseparable connection between RDW and inflammatory response (Fava et al., 2019; Jandaghian et al., 2021). The inflammatory response causes red blood cell maturation disorder through iron metabolism disorder and erythropoietin destruction, resulting in immature red blood cells into the bloodstream, but also reduce the survival rate of red blood cells, resulting in the mixing of red blood cell volume in circulation and other ways to change RDW (Salvagno et al., 2015). And some other studies have also shown that RDW is related to some conventional inflammatory markers such as C-reactive protein, erythrocyte sedimentation rate, tumor necrosis factor-α and interleukin-6 (He et al., 2018; Fava et al., 2019). In the CMA analysis, we have demonstrated that the beneficial effects of bronchoscopy on VAP patients are partially mediated by changes in RDW levels. Bronchoscopy is also essential for clinical targeted antimicrobial therapy to control inflammation, as it helps to obtain more accurate etiology, resulting in better diagnostic information. The findings of Christopher et al. showed that diagnostic bronchial therapy can help reduce the use of antibiotics and shorten the length of hospital stay (Guidry et al., 2014). Another retrospective study also indicated that the correct use of bronchoscopy can help intensive care clinicians formulate specific antibacterial treatments (Allen et al., 1994). Therefore, we can think that the reason why bronchoscopy can improve the prognosis of VAP patients is that whether it is aspiration of sputum or getting more accurate pathogens to help the adjustment of antibiotics, they all help to improve the inflammatory response of patients to some extent. Some scholars’ study has shown that early bronchoscopy is associated with a lower 90-days mortality rate in mechanically ventilated patients, which also supports our findings (Lee et al., 2015).

The results of the present subgroup analysis indicated no significant interaction between the subgroups regarding in-hospital mortality, indicating that whether the ICU patient population with different characteristics underwent bronchial examination was consistent with the in-hospital mortality risk. Among the outcomes for ICU mortality, after bronchoscopy, patients with MI or CPD had a lower mortality risk than those who did not undergo bronchoscopy. When bronchoscopy is performed on patients in the ICU without LD, the mortality risk was lower than for those with LD. We will explain it step by step, chronic obstructive pulmonary disease and bronchiectasis are common chronic lung diseases chronic obstructive pulmonary disease and bronchiectasis are common chronic lung diseases (Raherison and Girodet, 2009; Hill et al., 2019), such patients are prone to infection and inflammation due to dysfunctional airway clearance and defense, and long-term chronic inflammation leads to bronchial epithelial cell degeneration, necrosis, bronchial scarring, distortion, impaired lung function, and sputum accumulation, which are more likely to occur in the presence of acute inflammation (Tantucci and Modina, 2012; Hill et al., 2019). There is research evidence that the hemodynamic changes in patients with MI will lead to varying degrees of pulmonary function changes (Hales and Kazemi, 1977), and there is a pathophysiological basis for the interaction between some chronic lung diseases and MI (Goedemans et al., 2020). Therefore, we can consider that when VAP is present, bronchoscopy is more likely to benefit in patients with chronic inflammatory disease of the lungs and varying degrees of changes in lung function. The liver can synthesize and remove most coagulation factors, plasminogen, etc., and so LD can lead to coagulation and anticoagulation balance disorders (Amitrano et al., 2002; Monroe and Hoffman, 2009). One of the main complications of bronchoscopy is tracheal mucosal bleeding, so patients without LD are more likely to benefit from bronchoscopy than those with LD.

## Strengths and Limitations

This research study was the first to explore the entire patients with VAP in the ICU from MIMIC-IV database, the large data sample provides a solid basis for the results. Furthermore, PSM and IPTW further confirmed the results. We also found that alterations in RDW mediated part of the positive effect of bronchoscopy on the prognosis of VAP patients by CMA methods. The technology for bronchoscopy is currently quite mature, and the results of this study provide evidence to support the guidance of clinicians for using this technique. Of course, our research still had certain limitations. First of all, because the specific diagnosis time of patients is not recorded in the analyzed database, this study cannot determine the duration between bronchoscopy and VAP, only how bronchoscopy impacts the prognoses of patients with VAP who received mechanical ventilation. Because of this we were also unable to analyze the specific antibiotics for VAP treatment, and so we did not consider the impact of antibiotic use on the outcome. Second, although we adopted multivariate analysis to control for confounding factors that may affect the results as much as possible, there are still potential confounding factors that cannot be corrected in a retrospective study. Finally, the interaction between subgroups needs to be analyzed prospective in a larger sample of people for further confirmation.

## Conclusion

Bronchoscopy during MV was associated with reducing the risk of ICU and in-hospital mortality in patients with VAP in ICUs, and this beneficial effect was partially mediated by changes in RDW levels.

## Data Availability

Publicly available datasets were analyzed in this study. This data can be found here: The data were available on the MIMIC-IV website at https://mimic.physionet.org/, https://doi.org/10.13026/a3wn-hq05.

## Glossary

AST: aspartate aminotransferase
ALT: alanine aminotransferase
AP: alkaline phosphatase
APSIII: Acute Physiology Score III
AG: anion gap
BE: base excess
BUN: blood urea nitrogen
ACME: average causal mediating effect
ADE: average direct effect
BMI: body mass index
CRRT: continuous renal replacement therapy
CHF: congestive heart failure
CD: cerebrovascular disease
CPD: chronic pulmonary disease
LD: liver disease
CMA: causal mediation analysis
LDH: lactate dehydrogenase
IMV: invasive mechanical ventilation
ICU: intensive care unit
INR: international normalized ratio
KM: Kaplan-Meier
VIF: variance inflation factor
IPTW: propensity score-based inverse probability of treatment
MCV: mean corpuscular volume
MCH: mean corpuscular hemoglobin
MCHC: mean corpuscular hemoglobin concentration
MICU: Medical Intensive Care Unit
MIMIC: Medical Information Mart for Intensive Care
MI: myocardial infarction
mHR: mean heart rate
mMAP: mean value of mean arterial pressure
mRR: mean respiratory rate
mT: mean temperature
mSpO2: mean SpO2()
PT: prothrombin time
PTT: partial thromboplastin time
PSM: propensity score matching
RD: renal disease
RBC: red blood cells
RDW: red blood cell distribution width
SICU: Surgical Intensive Care Unit
VAP: ventilator-associated pneumonia
WBC: white blood cells
