# Supplementary figures and images for: The Association Between Bronchoscopy and the Prognoses of Patients With Ventilator-Associated Pneumonia in Intensive Care Units: A Retrospective Study Based on the MIMIC-IV Database
Source: Front Pharmacol. 2022 Jun 8;13:868920. doi: 10.3389/fphar.2022.868920 (PMC9214225; doi:10.3389/fphar.2022.868920)

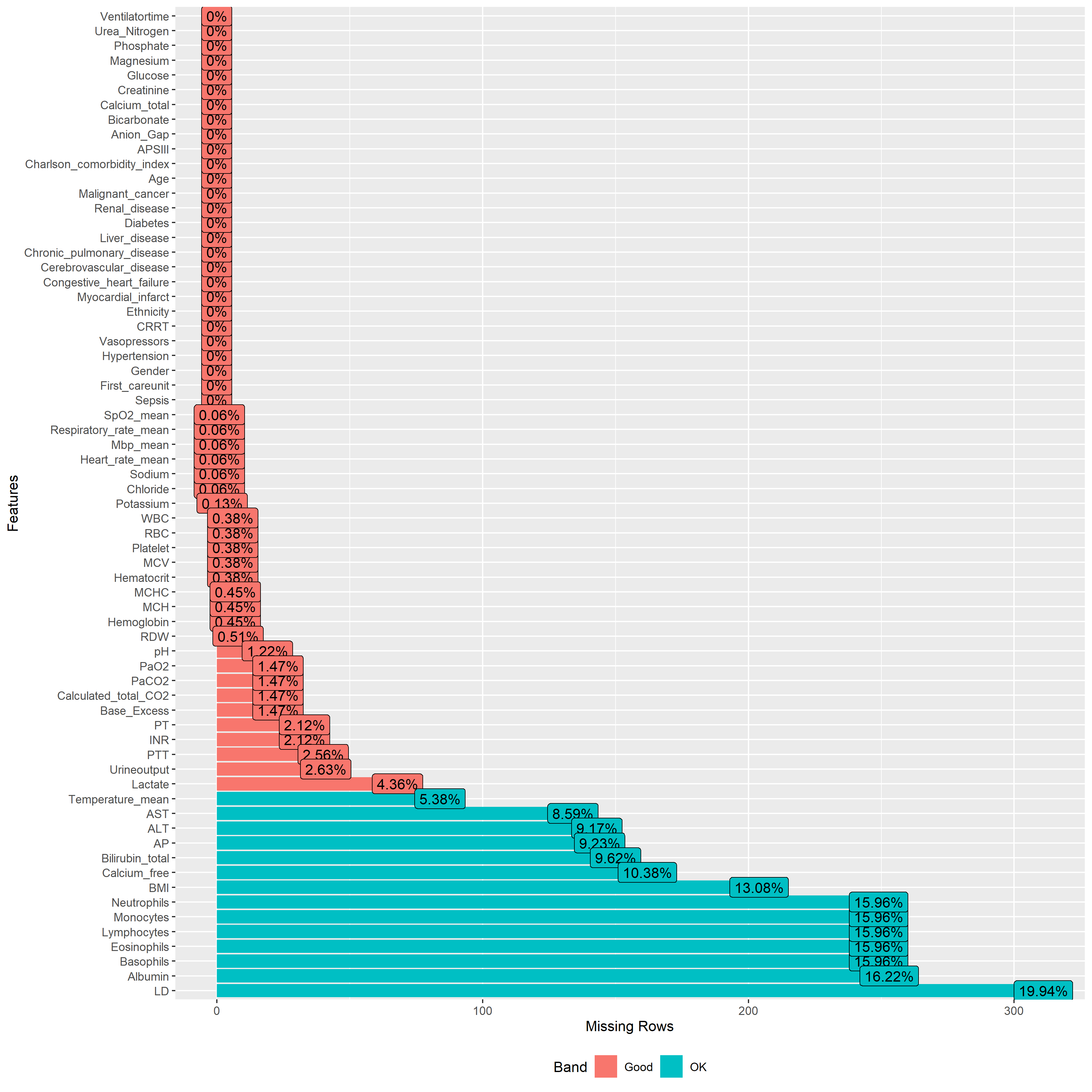

Supplement: Supplementary file 2 [file Image1.TIFF]

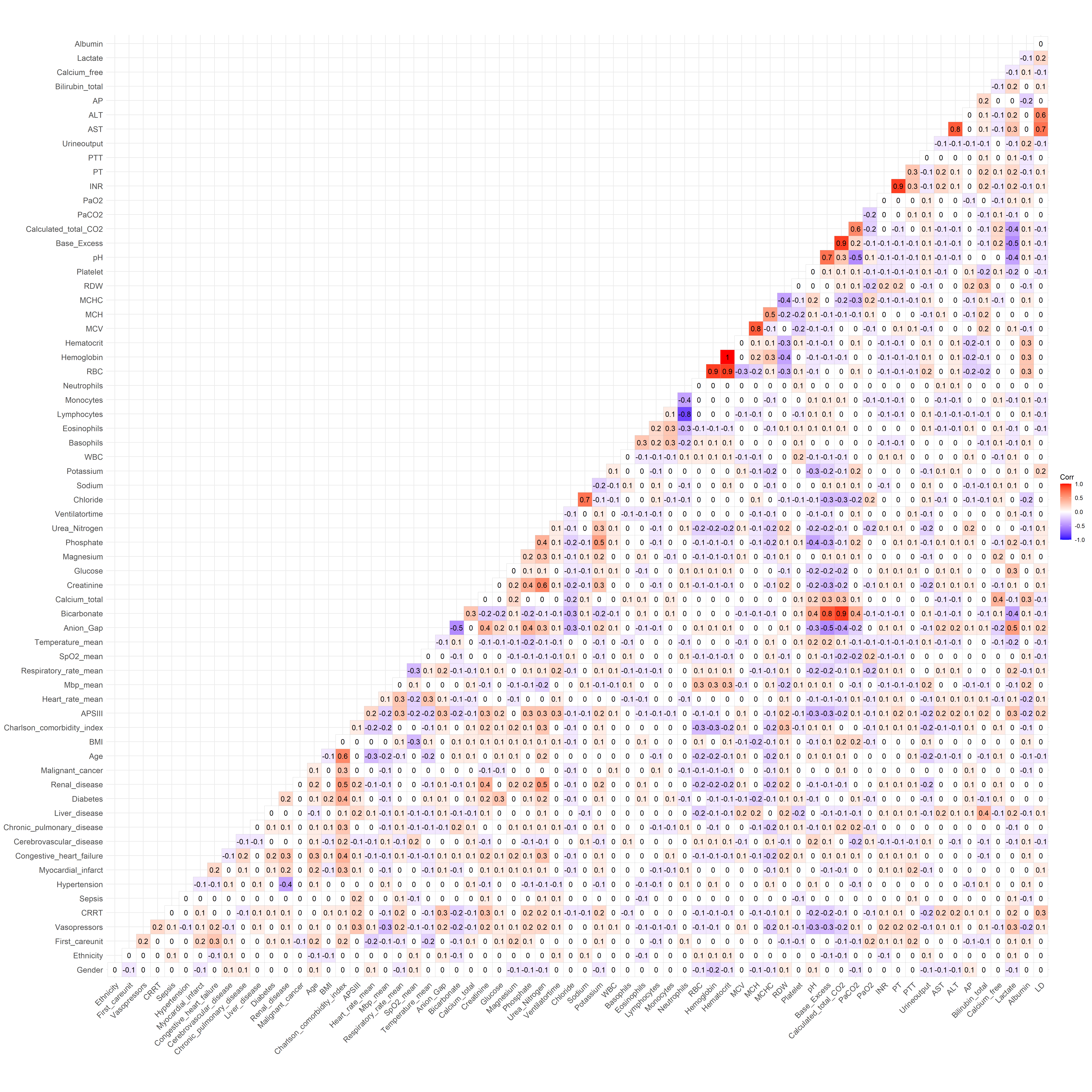

Supplement: Supplementary file 4 [file Image2.TIFF]
